# Impact of selected solvent systems on the pore and solid structure of cellulose aerogels

**DOI:** 10.1007/s10570-016-0896-z

**Published:** 2016-03-07

**Authors:** Nicole Pircher, Leticia Carbajal, Christian Schimper, Markus Bacher, Harald Rennhofer, Jean-Marie Nedelec, Helga C. Lichtenegger, Thomas Rosenau, Falk Liebner

**Affiliations:** Division of Chemistry of Renewable Resources, University of Natural Resources and Life Sciences Vienna, Konrad-Lorenz-Straße 24, 3430 Tulln, Austria; Institute of Chemistry of Clermont-Ferrand, Clermont Université, Ecole Nationale Supérieure de Chimie de Clermont-Ferrand, BP 10448, 63000 Clermont-Ferrand, France; UMR 6296, Institute of Chemistry of Clermont-Ferrand, Centre National de la Recherche Scientifique, 24 Avenue des Landais, 63171 Aubière, France; Institute of Physics and Material Sciences, University of Natural Resources and Life Sciences Vienna, Peter Jordan Straße 82, 1190 Vienna, Austria

**Keywords:** Cellulose aerogels, Cellulose solvents, Porous solids, Thermoporosimetry

## Abstract

The impact of selected cellulose solvent systems based on the principal constituents tetrabutylammonium fluoride (TBAF), 1-ethyl-3-methyl-1*H*-imidazolium-acetate, *N*-methylmorpholine-*N*-oxide, or calcium thiocyanate octahydrate (CTO) on the properties of cellulose II aerogels prepared from these solvent systems has been investigated as a means towards tailoring cellulose aerogel properties with respect to specific applications. Cotton linters were used as representative plant cellulose. Cellulose was coagulated from solutions with comparable cellulose content, and dried with supercritical carbon dioxide after solvent exchange. The resulting bulk aerogels were comprehensively morphologically and mechanically tested to relate structure and mechanical properties. Different solvent systems caused considerable differences in the properties of the bulk samples, such as internal surface area (nitrogen sorption), morphology, porosity (He pycnometry, thermoporosimetry), and mechanical stability (compression testing). The results of SAXS, WAXS, and solid-state ^13^C NMR spectroscopy suggest that this is due to different mechanisms of cellulose self-assembling on the supramolecular and nanostructural level, respectively, as reflected by the broad ranges of cellulose crystallinity, fibril diameter, fractal dimension and skeletal density. Both solid state NMR and WAXS experiments confirmed the sole existence of the cellulose II allomorph for all aerogels, with crystallinity reaching a maximum of 46–50 % for CTO-derived aerogels. Generally, higher fibril diameter, degree of crystallinity, hence increased skeletal density were associated with good preservation of shape and dimension throughout conversion of lyogels to aerogels, and enhanced mechanical stability, but somewhat reduced specific surface area. Amorphous, yet highly rigid aerogels derived from TBAF/DMSO mixtures deviated from this trend, most likely due to their particular homogeneous and nanostructured morphology.

**Electronic supplementary material:**

The online version of this article (doi:10.1007/s10570-016-0896-z) contains supplementary material, which is available to authorized users.

## Introduction

Aerogels are solids consisting of a coherent open-porous network of loosely packed, bonded cellulose particles or nanofibrils whose voids are filled with gases, such as air, and feature very low density and high specific surface area (Liebner et al. 2012a). Depending on the source material and preparation method, their solid structure can consist of the cellulose polymorphs I (Iα: dominating in bacterial cellulose; Iβ: principal crystal phase of native plant cellulose) or II, the sole allomorph of cellulose coagulated from solution state. In any case, intrinsic characteristics of the cellulosic source material, such as molecular weight average and distribution or purity—for example in terms of residual lignin content in wood pulp or content of carboxyl- and carbonyl groups—substantially govern the material properties of the derived aerogels. For cellulose II aerogels—the target material of this study—morphology and mechanical properties can be furthermore controlled by the type of solvent used to shape aerogels from solution state.

Cellulose solvents share the capability to disrupt and rearrange the complex intra- and inter-molecular hydrogen bond networks in the different cellulose allomorphs, thereby achieving cellulose solubility. Several solvents have previously been used for the preparation of cellulosic aerogels, including molten Ca(SCN)_2_·nH_2_O, an inorganic salt hydrate (Hoepfner et al. 2008; Jin et al. 2004), aqueous alkali hydroxides, such as aq. NaOH (Cai et al. 2008; Gavillon and Budtova 2008) or aq. LiOH (Cai et al. 2008) [often under addition of solubilizing additives such as urea (Cai et al. 2008) or thiourea (Zhang et al. 2012)], a range of low-melting organic salts (ionic liquids) (Aaltonen and Jauhiainen 2009; Sescousse et al. 2011), and the tertiary amine oxide *N*-methylmorpholine-*N*-oxide monohydrate (NMMO·H_2_O), which is used in the large-scale production of Lyocell fibers (Innerlohinger et al. 2006; Liebner et al. 2009). A comprehensive summary of cellulose solvents with focus on manufacture of cellulosic aerogels, including particularities regarding their cellulose dissolving performance, handling throughout the processing chain and state of knowledge regarding their impact on the morphology of the aerogels cellulose aggregate structure, can be found in the encyclopedia chapter *Cellulose*-*based aerogels* (Liebner et al. 2015).

While efforts regarding the characterization of cellulosic aerogels are commonly focusing on bulk density, pore volume, pore size distribution, response towards compressive stress, and putative internal surface morphology, as studied by ESEM or SEM on fragments taken from the interior of the aerogel by gentle plucking, there is no comprehensive study on the effect of different solutions on the solid structure, such as crystallinity, nanostructure etc.

In this work, the impact of different cellulose solvents on the properties of lightweight cellulose II aerogels (ρ < 70 mg cm^−3^) obtained from cotton linters as a representative source of plant cellulose, has been investigated with regard to bulk properties, pore features and cellulose aggregate structure. To this end, four cellulose solvent systems, namely mixtures of (1) tetrabutylammonium fluoride and DMSO (TBAF/DMSO), (2) 1-ethyl-3-methyl-1*H*-imidazolium acetate and DMSO ([EMIm][OAc]/DMSO), (3) calcium thiocyanate octahydrate and lithium chloride (CTO/LiCl), and (4) molten *N*-methylmorpholine-*N*-oxide monohydrate (NMMO·H_2_O), have been studied. While each of these cellulose solvents has certain pros and cons with regard to cellulose dissolution, coagulation and conversion of cellulosic lyogels to aerogels, calcium thiocyanate octahydrate (CTO) is considered the most promising solvent due to its low costs and environmental impact.

The morphology of the obtained fragile open porous ductile materials was studied by scanning electron microscopy (SEM). Small angle X-ray scattering (SAXS) was used to determine fractal dimension and average size of the cellulose fibrils forming the respective scaffolds. Cellulose crystallinity was determined by both wide-angle X-ray scattering (WAXS) and solid-state ^13^C NMR spectroscopy. Possible thermal and chemical degradation of cellulose induced by the different dissolution approaches was evaluated by size exclusion chromatography. Skeletal density of the cellulose nanofibrils was measured by helium pycnometry allowing for calculation of sample porosity. Thermoporosimetry analysis was used to obtain pore size distributions comprising the full nanometer and low micrometer range. Internal surface areas were calculated from nitrogen adsorption/desorption experiments at 77 K and SAXS studies.

The comprehensive study of the nanostructure and material properties of the cellulose II aerogels was carried out to evaluate the influence of the respective cellulose solvent system with the aim of tailoring aerogel properties for a wide range of promising applications.

## Materials

Calcium thiocyanate hydrate [Ca(SCN)_2_·*n*H_2_O] was synthesized from calcium hydroxide and ammonium thiocyanate (Pircher et al. 2015). *N*-benzylmorpholine-*N*-oxide (NBnMO), added as sacrificial scavenger of *carbenium iminium* ions, which autocatalytically decompose NMMO·H_2_O under cellulose dissolving conditions, was prepared according to a method described in an earlier work (Liebner et al. 2012b). Lithium chloride (LiCl), propyl gallate (PG) and tetrabutylammonium fluoride trihydrate (TBAF·3H_2_O) were purchased from Sigma-Aldrich (Vienna, Austria). Dimethyl sulfoxide (DMSO) was a product of Merck (Vienna, Austria). 1-Ethyl-3-methyl-1*H*-imidazolium acetate ([EMIm][OAc]) was a donation of BASF (Ludwigshafen, Germany). Absolute ethanol was purchased from Fisher Scientific (Vienna, Austria). *N*-methylmorpholine-*N*-oxide monohydrate (NMMO·H_2_O) was of technical grade. Cotton linters (CL, M_w_ 133.5 kg mol^−1^, CCOA 4.2 µmol g^−1^ C=O, FDAM 8.8 µmol g^−1^ COOH) were used as a cellulose source.

### Preparation of cellulose II lyogels using different cellulose solvent/anti-solvent systems

Prior to dissolution, cotton linters were activated by disintegration in deionized water using a kitchen blender for about 30 s and subsequent swelling in deionized water. For all solvent exchange steps sample-to-liquid volume ratios of 1:10 were maintained.

#### NMMO·H_2_O

Cellulose aerogels according to the Lyocell route were prepared by dissolving 3 w% CL in NMMO·H_2_O. 1 w‰ PG and 1 w‰ NBnMO were added to suppress homogeneous and heterogeneous side reactions (Rosenau et al. 2002, 2005). A visually clear cellulose solution was obtained after 20 min of vigorous stirring at 110 °C. The Lyocell dope was cast into cylindrical molds (Ø 10 mm, h 10 or 20 mm) and solidified upon cooling to room temperature. Cellulose coagulation was achieved by immersion of the samples in 96 % ethanol. Subsequently, leaching of the cellulose solvent including the stabilizers and simultaneous transfer to a solvent miscible with scCO_2_ was accomplished by immersion in absolute ethanol, which was replaced three times every 24 h.

#### Ca(SCN)_2_·8H_2_O/LiCl

1.5 or 2.0 w% CL were dissolved in a melt consisting of Ca(SCN)_2_, deionized water and 2.5 w% LiCl. Prior to heating of the solvent system, the actual water content of Ca(SCN)_2_ was determined by Karl–Fischer titration and adjusted to a molar ratio of 8:1 (H_2_O:Ca^2+^) corresponding to the highest coordination number of calcium ions (Hoepfner et al. 2008). Full dissolution of cotton linters in freshly prepared CTO/LiCl melt was achieved within 120 min of agitation at 140 °C, as confirmed by light microscopy (NOVEX Holland, B-Series, 200× magnification). Subsequently, vacuum was applied to remove air bubbles from the viscous cellulose solution.

The hot cellulose solution was cast into the above-described molds, cooled to room temperature and covered with an excess of 96 v% ethanol to coagulate the cellulose. The solidified bodies were subsequently transferred into aqueous 50 v% ethanol and then repeatedly immersed in deionized water until conductivity of the washing bath was below 1 µS. Prior to scCO_2_ drying, the samples were immersed in absolute ethanol, which was replaced two times every 6–12 h.

#### TBAF/DMSO

A stock solution was prepared by dissolving 315.12 g TBAF·3H_2_O in 684.88 g anhydrous DMSO (1 mol kg^−1^). The solvent mixture, which had a water content of 5.4 w% H_2_O (determined by Karl-Fischer titration), was dried by adding 650 g molecular sieve 4 Å (Sigma-Aldrich), until the water content was about 1.5 w% (TBAF·0.79H_2_O). Subsequently, the stock solution was diluted with anhydrous DMSO to obtain a TBAF content of 16.6 w% and a water content of 0.8 w%, respectively. For the preparation of cellulose solutions, the solvent mixture was pre-heated to 60 °C. 3 w% CL were added in portions and dissolved under gentle stirring for 24 h. Casting of the cellulose solution and coagulation of cellulose was accomplished in the same way as described for the Ca(SCN)_2_·8H_2_O/LiCl solvent system. However, washing with water was not required due to the good solubility of TBAF in ethanol. Instead, removal of the cellulose solvent and simultaneous transfer to a solvent miscible with scCO_2_ was accomplished by immersion of the samples in absolute ethanol, which was replaced three times every 24 h.

#### [EMIm][OAc]/DMSO

1.5 or 3.0 w% CL were dissolved in a mixture of the ionic liquid [EMIm][OAc] and DMSO (3:7, v/v) at room temperature under agitation. After 2 h, CL were completely dissolved as confirmed by light microscopy. After degassing and casting the solutions into respective molds, the respective cellulose was coagulated by addition of 96 % ethanol (*cf*. CTO/LiCl solvent system). The removal of the cellulose solvent was accomplished by immersion in the cellulose anti-solvent (96 % ethanol), which was replaced four times every 6–12 h. Prior to scCO_2_ drying, the samples were immersed in absolute ethanol, which was replaced two times every 6–12 h.

### Supercritical carbon dioxide drying

Extraction of the cellulose anti-solvent ethanol and conversion of the lyogels to aerogels was accomplished by supercritical carbon dioxide (scCO_2_) drying. The samples were placed into a 300 mL autoclave equipped with a separator for carbon dioxide recycling (Separex, France). Drying was performed under constant flow of scCO_2_ (40 g min^−1^) at 10 MPa and 40 °C for 2–3 h. The system was then slowly and isothermally depressurized at a rate of <0.1 MPa min^−1^.

### Characterization

#### Bulk density and shrinkage

Shrinkage of the shaped cellulose solutions and the resulting lyogels during the coagulation/solvent exchange and supercritical carbon dioxide drying steps was evaluated by following the respective volumetric changes of the specimen at the respective stages. To calculate bulk densities, the weight of the aerogels was determined gravimetrically.

#### Scanning electron microscopy

Scanning electron microscopy (SEM) of gold sputtered samples (Leica EM SCD005 sputter coater, layer thickness 6 nm) was performed on a Tecnai Inspect S50 instrument under high vacuum using an acceleration voltage of 5.00 kV.

#### Mechanical testing

Mechanical response profiles towards compressive stress were recorded on a Zwick-Roell Materials Testing Machine Z020. The required strain to achieve a deformation speed of 2.4 mm min^−1^ was measured with a 500 N load cell. Yield strength (R_P0.2_) was defined as the stress at 0.2 % plastic deformation.

#### Small and wide angle X-ray diffraction

X-ray scattering was performed on a Rigaku S-Max 3000 with MM002+ Cu-K_α_ source (wavelength λ = 0.154 nm). Scattering images were averaged over the azimuth to obtain information on the intensity I(q) depending on the reciprocal lattice vector q which is related to the scattering angle (2Θ), and the wavelength of the X-ray radiation (λ) (Eq. 1):

1 $$ {\text{q}} = 4\uppi \sin \left(\Theta  \right) /\uplambda. $$

The signals showed isotropic structure, i.e. no preferred orientation was detected for all materials. With the aim to access scattering vectors in range of 0.06–7 nm^−1^ (Small Angle X-ray Scattering, SAXS) and 5–25 nm^−1^, i.e. 8°–38° 2Θ (Wide Angle X-ray Scattering, WAXS), respectively, all samples were measured in different distances to the detector.

SAXS data were background-corrected and analyzed for the radius of cellulose nanofibrils forming the aerogel network and the fractal dimension (FD) of the network. Furthermore, the specific inner surface was calculated from the Porod constant (P), proportional to the pore surface (S), and by taking the scattering invariant (Inv) (Eq. 2) into account, which is proportional to the pore volume (V). The ratio P/Inv corresponds to the surface area-to-volume ratio of the pores in the sample volume irradiated by the X-ray beam (Eq. 3).

2 $$ {\text{Inv}} = \mathop \smallint \limits_{0}^{\infty } {\text{q}}^{2} {\text{I}}\left( {\text{q}} \right){\text{dq}} $$

3 $$ {\text{P/Inv}} = 1 /(\uppi \cdot\upphi_1 \cdot\upphi_2) \cdot {\text{S/V}} $$

where ϕ_1_ and ϕ_2_ = 1 − ϕ_1_ are the volume fractions of the pores and the cellulose material.

The results of WAXS were used to calculate cellulose crystallinity (Eq. 4) based on a comparison of the intensities of the main visible peak at about 20° (I_cryst_) and of a peak at a position, which is assumed to show scattering from amorphous phases only, such as that at 13.2 nm^−1^, i.e. 18° (I_amorph_) (Xu et al. 2013). Prior to integration all spectra were baseline-corrected.

4 $$ {\text{Crystallinity}}_{\text{WAXS}} = \frac{{{\text{I}}_{\text{cryst}} - {\text{I}}_{\text{amorph}} }}{{{\text{I}}_{\text{cryst}} }} \cdot 100\;\% $$

#### Solid state nuclear magnetic resonance

All solid state nuclear magnetic resonance (NMR) experiments were performed on a Bruker Avance III HD 400 spectrometer (resonance frequencies of ^1^H of 400.13 MHz and ^13^C of 100.61 MHz, respectively), equipped with a 4 mm dual broadband CP-MAS probe. ^13^C spectra were acquired using the TOSS (total sideband suppression) sequence, spinning rate 5 kHz, cross-polarization (CP) contact time 2 ms, recycle delay 2 s, SPINAL-64 ^1^H decoupling and acquisition time 43 ms. 2 k data points were sampled to give a spectral width of 240 ppm. Chemical shifts were referenced externally against the carbonyl signal of glycine (δ 176.03 ppm). The acquired FIDs were apodized with an exponential function (lb = 11 Hz) prior to Fourier transformation. Peak fitting was performed with the Dmfit program (Massiot et al. 2002).

#### Size exclusion chromatography

Size exclusion chromatography (SEC) on a system comprising four columns (PLgel MIXED-A LS, 20 µm, 7.5 × 300 mm), multiple-angle laser light scattering (MALLS) and refractive index (RI) detection, was performed to determine the molecular weight distribution of cellulose prior to and after processing to aerogels. The respective samples were activated using the standard method involving successive immersion in H_2_O, ethanol and DMAc and subsequently dissolved in *N*,*N-*dimethylacetamide (DMAc)/LiCl (9 % w/v). A mixture of DMAc/LiCl (0.9 %w/v) was used as SEC mobile phase (Röhrling et al. 2002). Calculations were performed using a dn/dc value of 0.136 ml g^−1^ (Schelosky et al. 1999).

#### Thermoporosimetry

An important characteristic of cellulosic aerogels is their hierarchical pore structure which co-determines their fascinating properties. However, it is not easy to analyze the entire pore size distribution (PSD) with only a single technique. Commonly, the PSD of aerogels is studied using mercury intrusion and nitrogen sorption methods. N_2_ sorption however only covers pore sizes of up to about 170 nm, which is insufficient for the broad PSDs inherent to most cellulose-based aerogels (single-digit nano- to low micrometer range). Mercury intrusion is not an appropriate technique to characterize the fragile, ultra-lightweight cellulose aerogels, as the nanofibrillary network of soft matter is inevitably destroyed by this method. Therefore, the PSD can only be calculated from the bulk volume reduction (sample compression) rather than from intrusion of mercury into the pore system, which in fact does not occur (non-intrusive mercury porosimetry). After applying a certain mercury pressure, the PSD below the largest pore size affected by the densification can be characterized by nitrogen sorption experiments (Pirard et al. 2003; Rudaz et al. 2014).

Thermoporosimetry (TPM), a method relying on the Gibbs–Thomson equation (Eq. 5), has been proposed as an alternative technique to characterize the structure of porous aerogels (Gibbs 1928; Thomson 1872). The Gibbs–Thomson equation quantifies the experimental shift of the melting point of an interstitial liquid caused by its confinement in small pores and can be written as:

5 $$ \Delta {\text{T}} = {\text{T}}_{\text{P}} - {\text{T}}_{0} = \frac{{2\upsigma_{\text{SL}} \cos\uptheta{\text{T}}_{0} }}{{\Delta {\text{H}}_{\text{m}}\uprho_{\text{s}} {\text{R}}_{\text{p}} }}\sim\frac{\text{k}}{{{\text{R}}_{\text{p}} }} $$

where T_P_ is the melting temperature of a liquid confined in a pore of radius R_p_, T_0_ is the normal melting temperature of the liquid, σ_SL_ is the surface energy of the solid/liquid interface, θ is the contact angle, ΔH_m_ is the melting enthalpy and ρ_S_ is the density of the solid.

According to this equation, the shift of the transition temperature of a confined liquid ΔT is inversely proportional to the radius of the pore in which it is confined. It is, however, well known that not the entire solvent takes part in the transition and a significant part remains adsorbed onto the surface of the pore. The state of this adsorbed layer has been discussed extensively for the case of water. Consequently, the radius in the Gibbs–Thomson equation should be written as R = R_p_ − t where t is the thickness of the adsorbed layer leading to a reformulation of Eq. (5) as Eq. (6):

6 $$ {\text{R}}_{\text{P}} = \frac{\text{k}}{{\Delta {\text{T}}}} + {\text{t}} $$

In principle, it is then possible to determine the pore size of a given material by measuring ΔT. Kuhn et al. (1955) proposed to use differential scanning calorimetry to measure ΔT and invented the so-called “thermoporometry” technique (or “thermoporosimetry” as will be used in the following). This technique has been further described by Fagerlund (1973) and popularized and developed by Brun et al. (1977). Knowledge of k and t in Eq. (6) is mandatory, but once determined, the curve obtained from TPM can be transformed into the PSD. In this sense, TPM is a secondary method since it requires preliminary determination of the evolution of ΔT as a function of R_p_ for a given solvent.

Based on previous studies investigating the applicability of *o*-xylene as a TPM solvent to determine the pore size distribution of silica aerogels (Dessources et al. 2012), the following equation was derived:

7 $$ {\text{R}}_{\text{P}} = {\text{t}}\exp \left( {\frac{ - 1}{{{\text{c}}\Delta {\text{T}}}}} \right) $$

where t = 1.4919 nm is the thickness of the layer of the solvent remaining adsorbed onto the surface of the pores and c = 0.03706 °C^−1^.

This mathematical model avoids simplification of the hypothesis and has proven to be the most suitable for this solvent in particular for the high value of R_p_ inherent to cellulosic aerogels (≤10 µm) (Billamboz et al. 2005).

Variation of solvent crystallization enthalpy as a function of temperature has to be taken into account for a correct calculation of PSD (Brun et al. 1977). Knowing the porous volume of the reference silica gels, this can be accomplished by considering the amount of *o*-xylene undergoing phase transition. The following dependence was found (Eq. 8):

8 $$ {\text{W}}_{\text{a}} = {\text{W}}_{0} \exp \left( {\frac{{\Delta {\text{T}}}}{\text{f}}} \right) $$

where W_o_ = 97.7 J cm^−3^ is the energy of crystallization of the bulk solvent and f = 57.9 °C.

Considering Eqs. (7) and (8) derived for mesoporous systems, PSDs can be determined according to Eq. (9):

9 $$ \frac{{{\text{dV}}_{\text{P}} }}{{{\text{dR}}_{\text{P}} }} = \frac{{{\text{Y}}\left( {\text{T}} \right)\Delta {\text{T}}^{2} {\text{c}}}}{{{\text{W}}_{\text{a}} {\text{R}}_{\text{P}} }} $$

where Y(T) is the heat flow of the sample given by the ordinate of the DSC thermogram.

In course of this work thermoporosimetry analysis was performed using a Mettler-Toledo DSC 823e differential scanning calorimeter (DSC) equipped with a liquid nitrogen module to extent the scanning range to −150–600 °C. Data processing was accomplished with the STARe software package. The DSC instrument was calibrated for both, temperature and enthalpy using open-porous metallic standards (In, Pb, Zn).

About 1–5 mg of the respective aerogel was weighed into a 160 µl aluminum DSC pan and soaked with *o*-xylene in slight excess to ensure both quantitative filling of the aerogels voids and presence of some free solvent to allow the measurement of its crystallization upon cooling. Crystallization curves were used instead of melting curves, but reversibility of the transitions was confirmed by cycling the system. All measurements were carried out in ambient atmosphere and included the following steps:

- Cooling from 25 °C down to −70 °C at a rate of 0.7 °C min^−1^;
- Heating from −70 °C up to T, at a rate of 0.7 °C min^−1^ (−30 °C ≤ T ≤ −20 °C);
- Cooling from T down to −70 °C at a rate of 0.7 °C min^−1^ and
- Final heating from −70 °C up to 25 °C at a rate of 0.7 °C min^−1^.

A slow rate of 0.7 °C min^−1^ was chosen to allow for continuous thermal equilibrium inside the sealed DSC cell. A total of 11 thermograms was acquired for each sample.

True skeleton densities (ρ_S_) of the aerogels were indirectly determined from the respective bulk densities (ρ_B_) of the samples and their porosity, as measured by helium gas pycnometry (Micromeritics Accupyc II 1340). He pycnometry (27 °C) was conducted 400 times per sample and the values were checked for stability (Incertitudes < 0.3 %).

Nitrogen adsorption/desorption isotherms were recorded at 77 K on a Quantachrome Autosorb1 instrument. All samples were degassed in vacuum prior to analysis. Specific surface areas were calculated from 11-point measurements using the Brunauer, Emmett and Teller (BET) equation.

## Results and discussion

### Impact of solvent on shrinkage and bulk density

The dimensional stability of cellulosic lyogels and aerogels along their preparation path and during storage depends on their solid content (which in turn mainly depends on the cellulose concentration of the respective solution), the particular cellulose aggregate structure (depending on the phase separation mechanism leading to cellulose coagulation) and the presence of hydrophilic cellulose solvent remnants, confined in fibrillary interstices of the lyogel or attached to cellulose. Traces of NMMO, for example, can result in adsorption and condensation of considerable amounts of water, which would inevitably lead to pore collapsing and shrinkage during scCO_2_ drying and sample storage (Liebner et al. 2009). Similarly, the derivatization of the reducing ends of cellulose by 1,3-dialkyl-1*H*-imidazolium ionic liquids will introduce hydrophilic functions as well, which might translate into modified bulk properties (Ebner et al. 2008).

The bulk density was found to be 30–40 mg cm^−3^ for aerogels derived from 1.5 w% solutions of CL and 55–67 mg cm^−3^ for 3 w% solutions. The observed variances within the two density levels are due to different shrinkage values caused by the mechanisms of cellulose coagulation associated to the respective cellulose solvents (*cf*. Morphology). The extent of shrinkage observed during the cellulose coagulation and solvent exchange (lyogel stage), as well as scCO_2_ drying steps (aerogel stage) is listed in Table 1.

**Table 1:** Bulk density (ρ_B_) and shrinkage during coagulation, solvent exchange (S_coag/exch_) and scCO_2_ drying (S_dry_) steps and total shrinkage (S_tot_) of aerogels derived from solutions of cotton linters (CL) in TBAF/DMSO (CL-TBAF), [EMIm][OAc]/DMSO (CL-EMIm), NMMO·H_2_O (CL-NMMO) and Ca(SCN)_2_·8H_2_O/LiCl (CL-CTO)

Sample name	CL-TBAF	CL-EMIm	CL-EMIm	CL-NMMO	CL-CTO	CL-CTO
CL (w%)	3.0	1.5	3.0	3.0	1.5	2.0
ρ_B_ (mg cm^−3^)	67.1 ± 3.3 (7)	40.2 ± 3.5 (7)	55.3 ± 1.4 (5)	62.9 ± 3.0 (9)	29.9 ± 0.8 (7)	48.2 ± 2.7 (6)
S_coag/exch_ (%)	20 ± 4 (7)	18 ± 7 (7)	9 ± 6 (5)	11 ± 5 (9)	17 ± 2 (7)	24 ± 3 (6)
S_dry_ (%)	19 ± 3 (7)	42 ± 2 (7)	26 ± 3 (5)	24 ± 3 (9)	7 ± 2 (7)	10 ± 2 (6)
S_tot_ (%)	35 ± 5 (7)	52 ± 5 (7)	33 ± 6 (5)	33 ± 2 (9)	23 ± 3 (7)	31 ± 4 (6)

The respective number of samples is given in brackets

Generally, a higher cellulose concentration (2–3 w%) resulted in a total shrinkage of about 30–35 %, independently of the solvent system. However, the contributions of the individual preparation steps varied strongly. For the CL-CTO system, volume-loss was highest at the lyogel formation stage (17–24 %) for both of the studied density levels. Since visually no shrinkage was observed during coagulation, it can be attributed to the solvent exchange, most likely the transfer from ethanol 96 v% to water. In accordance with an earlier study on CTO-derived cellulose aerogels (Hoepfner et al. 2008), surprisingly good volumetric preservation of the gels during scCO_2_ drying was observed (7–10 % shrinkage only). CL-EMIm and CL-NMMO aerogels prepared from 3 w% solutions of CL exhibited the opposite behavior: volume loss was low in the lyogel stage (9–11 %), but comparatively high during drying (24–26 %). The differences regarding shrinkage during scCO_2_ drying were particularly distinct for samples derived from low concentrations of CL (1.5 w%): the sixfold shrinkage (42 %) was observed for largely (or entirely) amorphous CL-EMIm aerogels (cellulose II crystallinity 0–15 %), when compared to CL-CTO samples (cellulose II crystallinity 46–50 %). However, as apparent from the results of compression testing (*cf*. Mechanical properties), differences in sample stability cannot be correlated exclusively to the crystallinity of the respective cellulose II networks, but are also strongly controlled by morphological features on nano- and microscale.

### Morphology

**Fig. 1:**
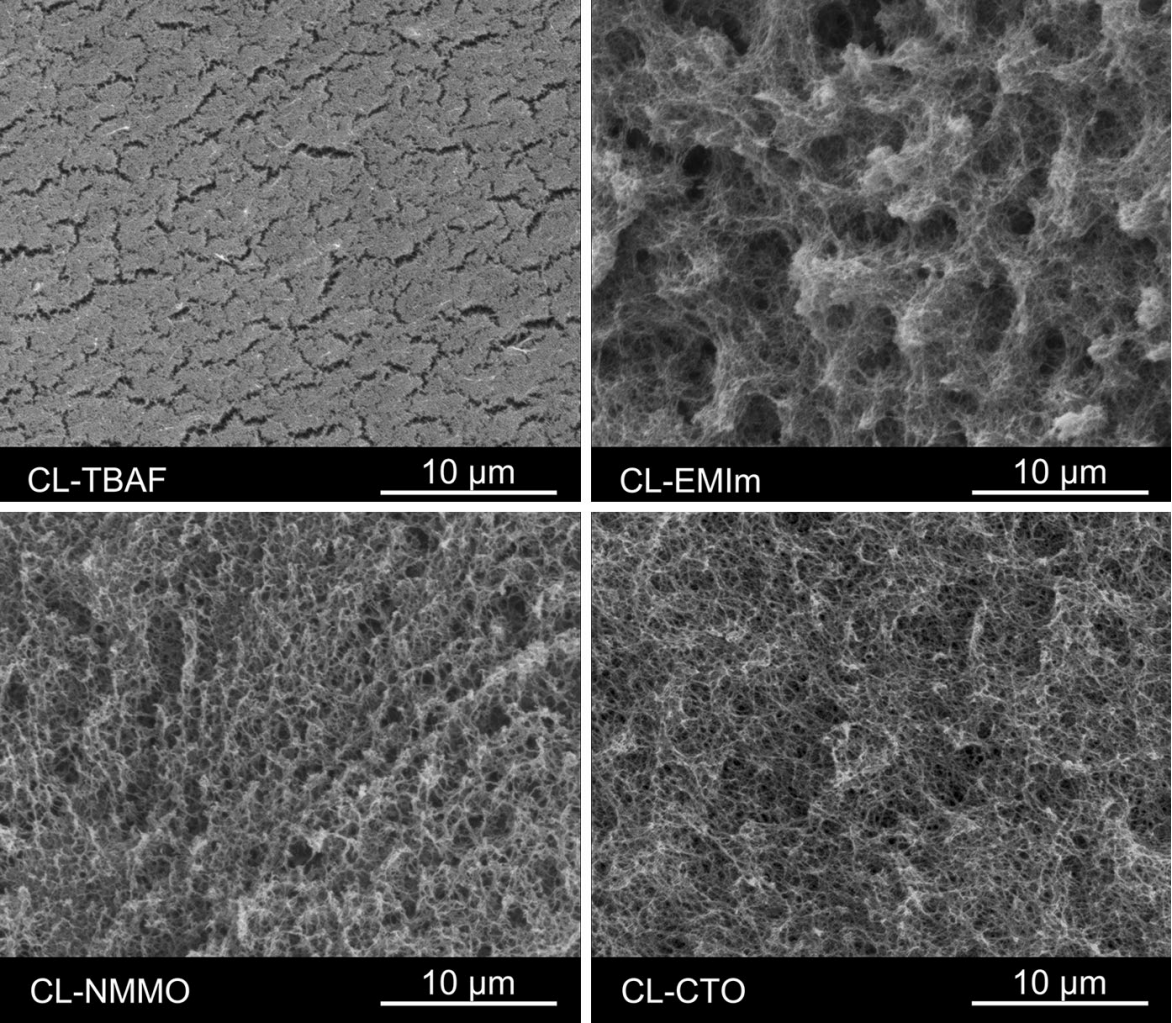
Scanning electron micrographs of aerogels derived from solutions of 3.0 w% cotton linters in TBAF/DMSO (CL-TBAF), [EMIm][OAc]/DMSO (CL-EMIm), NMMO·H2O (CL-NMMO) and 1.5 w% cotton linters in Ca(SCN)_2_·8H_2_O/LiCl (CL-CTO)

To visualize the internal surface morphology of the prepared aerogels largely free of structural artefacts arising from opening the samples, we cut the cylinders to a certain depth with a razor blade and thereafter carefully exposed a small piece of the aerogel body. The ripping edge of this sample was then sputter-coated with gold and analyzed by SEM (Fig. 1).

Depending on the respective solvent system, molecular disperse cellulose solutions can undergo phase separation upon standing, cooling, solidification, or addition of a cellulose antisolvent following different mechanisms. The morphology of the obtained aerogels is strongly influenced by these processes, as demonstrated by SEM in earlier studies for the cellulose solvent systems NMMO·H_2_O, [EMIm][OAc], [BMIm][Cl], and aqueous NaOH. This has been confirmed by the current study which extended the set of studied cellulose solvent systems to TBAF/DMSO and Ca(SCN)_2_·8H_2_O/LiCl. Different from earlier studies that were mainly based on low-molecular (M_W_ 29.2 kg mol^−1^), microcrystalline cellulose (Gavillon and Budtova 2008; Sescousse et al. 2011), this study employed non-degraded plant cellulose (cotton linters) of considerably higher molecular weight (M_W_ 133.5 kg mol^−1^). Beyond that, an investigation of the solid network morphology of the coagulated cellulose by SAXS, WAXS, and solid-state NMR spectroscopy was included.

Coagulation of cellulose from [EMIm][OAc]/DMSO yields networks of short nanofibers which assemble to globular superstructures, as illustrated in Fig. 1 (CL-EMIm). This morphology is assumed to be the result of a spontaneous one-step phase separation resembling spinodal decomposition, a mechanism which has been proposed for the coagulation of cellulose from respective solutions in [EMIm][OAc] or molten NMMO, both leading to networks of loosely agglomerated cellulose spheres (Gavillon and Budtova 2008; Sescousse et al. 2011). In case of TBAF/DMSO, the same mechanism of diffusion-controlled coagulation and gel formation, mediated through gradual replacement of fluoride anions by water (hydrated hydroxide ions) and increasing hydrogen bonding (Östlund et al. 2009), results in dense networks of very small interwoven nanofibers (CL-TBAF), and interconnected nanopore systems, which was confirmed by the small fibril and pore radii determined by SAXS and thermoporosimetry, respectively for this sample. The surface of ripping edge had a very flat appearance and could be regarded highly homogeneous, except for the high aspect ratio voids having a longitudinal dimension in the single-digit micrometer range. These fractures are likely caused by tensions affecting the cellulose network during coagulation and are reflected by a bimodal pore size distribution centered at 36 and 101 nm as determined by thermoporosimetry. In general solutions in TBAF and EMIm allow for the preparation of largely amorphous aerogels.

In contrast, SEM analysis of aerogels from solutions of cotton linters in NMMO·H_2_O (CL-NMMO) or Ca(SCN)_2_·8H_2_O/LiCl (CL-CTO) revealed rather random networks of cellulose nanofibrils. For melts of the NMMO and CTO solvent systems phase separation occurs in two steps. Already during cooling of the melt to room temperature, microphase separation of free and bound solvent, accompanied by cellulose alignment to larger nanofibrils, sets in. While the free solvent is easily removed upon addition of a suitable cellulose antisolvent, contact of the antisolvent with bound solvent causes additional cellulose coagulation, which happens in close proximity to the ordered cellulose domains formed during primary phase separation (Gavillon and Budtova 2008; Hoepfner et al. 2008). The comparatively slow phase separation seems to enable better orientation and assembling of cellulose as evident from the high cellulose II crystallinities of CL-CTO aerogels (*cf*. Solid characteristics) which is supposedly the main reason for the surprisingly low shrinkage of CTO-derived gels during conversion to aerogels. The relatively strong shrinkage of CL-NMMO samples (comparable with CL-EMIm) is assumed to be due to the particular solidification behavior of Lyocell dopes containing less than 6 w% of cellulose. Below 30 °C, NMMO of low cellulose content contracts significantly (Liu et al. 2001), which inevitably leads to cracks and voids inside the final aerogel bodies (Schimper et al. 2011).

### Mechanical properties

**Fig. 2:**
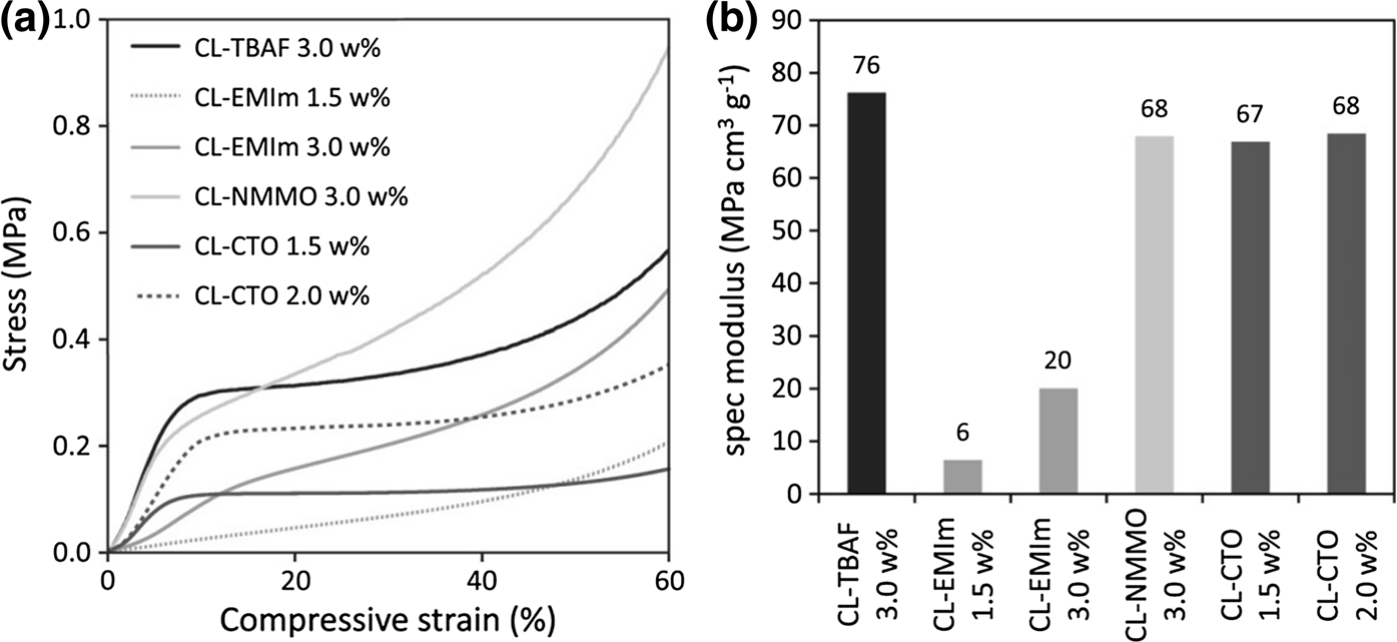
Compression tests: stress–strain curves (**a**) and specific moduli (**b**) of aerogels derived from solutions of cotton linters in TBAF/DMSO (CL-TBAF), [EMIm][OAc]/DMSO (CL-EMIm), NMMO·H_2_O [CL-NMMO, data obtained from (Liebner et al. 2009)] and Ca(SCN)_2_·8H_2_O/LiCl (CL-CTO)

The response of cellulosic aerogels towards compressive stress corresponds to Gibson and Ashbys *Mechanics of three*-*dimensional cellular* materials (Gibson and Ashby 1982) and has previously been discussed e.g. for bacterial cellulose aerogels (Liebner et al. 2012a). While the same general response regions can be assigned to all samples analyzed in this work, it is evident that the specific curve progression is significantly influenced by the applied solvent systems (Fig. 2a). For CL-TBAF, CL-NMMO and CL-CTO aerogels, a strong stress increase in the region of linear elastic deformation at low compressive strain entails higher elastic moduli (*E*_compr_), hence rigidity, compared to aerogels derived from solutions of CL in [EMIm][OAc]/DMSO (CL-EMIm). In contrast to the uniform nanostructure of CL-TBAF aerogels and the ordered crystalline fibrillary networks of CL-NMMO and CL-CTO, the comparatively heterogeneous morphology of CL-EMIm aerogels renders them very ductile.

When cell wall buckling [referring to the model introduced by Gibson and Ashby (1982)], hence non‐linear elastic deformation of the aerogel sets in, the slope of the curves flattens to a certain extent. The following plateau region between about 15 and 40 % strain indicates progressing plastic deformation due to cell collapsing (Gibson and Ashby 1982). The response profiles of CL-TBAF and CL-CTO aerogels show very distinct plateau regions, where energy is absorbed by the materials without a considerable increase in stress. In case of CL-EMIm and CL-NMMO aerogels, the stress gradually increases, indicating a higher load-bearing capacity during plastic deformation.

To account for the strong dependency of the mechanical properties of cellulosic aerogels on even small differences in their bulk density, respective density-normalized specific moduli and yield strength values (Table 2) have been calculated, which are more suitable to compare the lightweight samples prepared in this study differing in both structure and solid content.

**Table 2:** Mechanical properties under compressive stress: Young’s modulus (*E*), specific modulus (*E*_ρ_), yield strength (σ) and specific yield strength (σ_ρ_) of aerogels derived from solutions of cotton linters in TBAF/DMSO (CL-TBAF), [EMIm][OAc]/DMSO (CL-EMIm), NMMO·H_2_O (CL-NMMO) and Ca(SCN)_2_·8H_2_O/LiCl (CL-CTO)

Sample name	CL-TBAF	CL-EMIm	CL- EMIm^a^	CL-NMMO^b^	CL-CTO^a^	CL-CTO
CL (w%)	3.0	1.5	3.0	3.0	1.5	2.0
*E* (MPa)	5.12 ± 1.48 (7)	0.26 ± 0.02 (6)	1.12 ± 0.21 (5)	4.26	2.00 ± 0.07 (5)	3.30 ± 0.63 (5)
*E*_ρ_ (MPa cm^3^ g^−1^)	76	6	20	68	67	68
σ (kPa)	240 ± 14 (7)	22 ± 4 (6)	115 ± 11 (5)	184	76 ± 2 (5)	160 ± 23 (5)
σ_ρ_ (MPa cm^3^ g^−1^)	3.6	0.5	2.1	2.9	2.6	3.3

The respective number of samples is given in brackets

^a^Data obtained from Pircher et al. (2015)

^b^Data obtained from Liebner et al. (2009)

Cellulose aerogel networks containing a higher amount of ordered domains (CL-NMMO and CL-CTO aerogels) had similar specific moduli of 67–68 MPa cm^3^ g^−1^ (Fig. 2b). In contrast, the largely amorphous CL-TBAF and CL-EMIm aerogels showed significant differences in their rigidity. While the rather uniform morphology, fine fibrils and small pore size of CL-TBAF resulted in a brittle structure (which also explains the very smooth ripping edge observed upon SEM analysis) that proved to be very rigid under compressive stress, the open and more heterogeneous cellulose II network of CL-EMIm aerogels was very ductile compared to all other materials and showed a strong density-dependency compared to CL-CTO aerogels. The stress at which plastic deformation sets in (yield strength) shows similar trends, but a higher density dependency for both amorphous CL-EMIm as well as crystalline CL-CTO specimen (Table 2).

### Solid characteristics

#### Crystallinity and solid state structure

Solid state NMR analysis of the cellulosic raw material (cotton linters), gave a cellulose I crystallinity index of 68 %. Aerogels derived from all four solvent systems exhibited—if at all—exclusively cellulose II crystallinity.

**Fig. 3:**
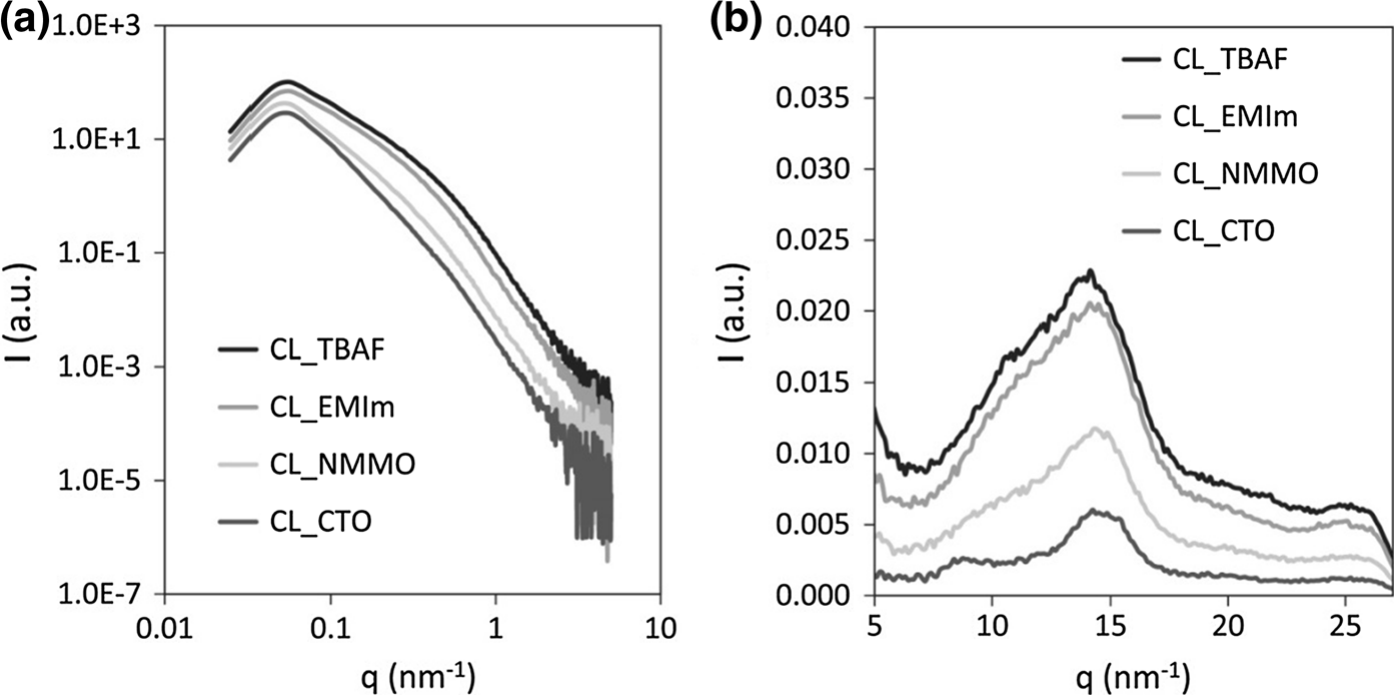
SAXS (**a**) and WAXS (**b**) patterns of aerogels derived from solutions of 3.0 w% cotton linters in TBAF/DMSO (CL-TBAF), [EMIm][OAc]/DMSO (CL-EMIm), NMMO·H_2_O (CL-NMMO) and 1.5 w% cotton linters in Ca(SCN)_2_·8H_2_O/LiCl (CL-CTO). Note that the intensity is in arbitrary units and does not reflect the degree of crystallinity, which is determined by the ratio of crystalline to amorphous signals

Coagulation of cellulose from solutions in TBAF or EMIm occurs by spinodal decomposition. This describes a spontaneous phase separation mechanism solely driven by diffusion which separates a solution of two or more components into distinct regions (or phases) that distinctly differ in both chemical composition and physical properties. The spontaneous materialization of a periodic variation of composition with distance as described by the Cahn–Hillard equation is assumed to be main reason for the low cellulose II crystallinities of samples CL-TBAF and CL-EMIm. Crystallinity for these samples was only indicative from the WAXS experiments (≤15 %; *cf*. Table 3) while NMR results suggest the absence of any cellulose II crystallinity. The patterns of small- and wide-angle X-ray scattering shown in Fig. 3 furthermore reveal higher cellulose II crystallinity for the aerogel samples CL-NMMO and CL-CTO whose cellulose networks had been formed by (1) micro phase separation of free and bound solvent fractions and (2) subsequent removal of both phases by a cellulose anti-solvent. This is most likely due to the comparatively slow first step that better allows for the formation of ordered cellulose domains. However, despite the concordant results of X-ray scattering and solid-state NMR, the comparatively low cellulose II crystallinity of CL-NMMO has to be treated with care as the respective WAXS diffractogram is equivalent to that of ball-milled cellulose (Nam et al. 2016) that can be fitted with a 9° pwhm cellulose II pattern and that corresponds to a crystallite size (Scherrer formula) of just a couple of cellulose chains, i.e. not a crystal in most views. Thus, CL-CTO can be regarded the only type of aerogel in this study that has considerable cellulose II crystallinity. Calculation of WAXS crystallinity using a different position, such as that at 11 nm^−1^ results in somewhat higher crystallinity values which diverge more from NMR crystallinities, however, without altering the afore-discussed trend amongst the different types of aerogels.

**Table 3:** Cellulose II crystallinity, cylinder radius (R_c_), porod exponent (PE) and skeletal density (ρ_S_) of aerogels derived from solutions of cotton linters (CL) in TBAF/DMSO (CL-TBAF), [EMIm][OAc]/DMSO (CL-EMIm), NMMO·H_2_O (CL-NMMO) and Ca(SCN)_2_·8H_2_O/LiCl (CL-CTO)

Sample name	CL-TBAF	CL-EMIm	CL-NMMO	CL-CTO
CL (w%)	3.0	3.0	3.0	1.5
Crystallinity (WAXS)	15	13	24	46
Crystallinity (NMR)	0	0	27	50
R_c_ SAXS (nm)	4.0 ± 0.1	4.3 ± 0.1	4.5 ± 0.1	4.5 ± 0.1
PE	1.9	1.9	2.4	3.0
ρ_S_ (g cm^−3^)	1.529 ± 0.002	1.527 ± 0.002	1.531 ± 0.002	1.543 ± 0.001

Pre-cooled aqueous solutions of alkali hydroxides represent another type of cellulose solvent that has recently been investigated for the preparation of aerogels. Similar as with NMMO·H_2_O or molten Ca(SCN)_2_·8H_2_O, phase separation occurs in two steps, affording cellulose II fibril networks of considerable crystallinity. Dissolution of cotton linters (74 % cellulose I crystallinity), for example, in an aqueous solution of 6 w% NaOH and 4 w% urea (pre-cooled and slurried at 4 °C, dissolution at −5 °C for 5 h), followed by cellulose coagulation with 2 % H_2_SO_4_ and exhaustive washing, resulted in a cellulose II crystallinity of 51 % (Zhang et al. 2001), which is comparable to that of the CL-CTO samples.

The skeletal or true density (ρ_S_) of all prepared aerogels was between 1.527 and 1.543 g cm^−3^ and increased with cellulose crystallinity (Table 3). In native cellulose, the density of a perfect crystal is 1.582 (cellulose Iα) and 1.599 g cm^−3^ (cellulose Iβ), respectively (Sugiyama et al. 1991). With an increasing amount of amorphous regions, hence less dense packing of cellulose molecules, the skeletal density of cellulose microfibrils decreases. Commercially available microcrystalline cellulose, such as Avicel^®^ PH-102 (cellulose I crystallinity >70 %), for example, has a true density of around 1.58 g cm^−3^ (Kumar et al. 2002; Rojas et al. 2011).

The size of the network-forming cellulose particles was determined by SAXS. Due to the fact that linear cellulose molecules aggregate into fibrillar entities, we generally assumed elongated fibrillary structures building up the solid aerogel matrices. Therefore the evaluation approach proposed by Jeong et al. (2005) for fibrillary structures, corresponding to cylinders longer than the low-q range of the SAXS measurement, was applied. The cylinder radius R_c_ was calculated by a modified Guinier fit yielding a radius of gyration R_g_ which is related to R_c_ by ${\text{R}}_{\text{c}} = \sqrt 2 {\text{R}}_{\text{g}}$. It was found that the mean radius of the cellulose fibrils was 4.0–4.5 nm for all aerogels investigated, but smallest for aerogels derived from solutions of CL in TBAF/DMSO (CL-TBAF), which showed a comparatively homogeneous and dense cellulose nanofibril structure (*cf*. Morphology) and the highest rigidity (*cf*. Mechanical properties) as well as surface area compared to the other samples.

From the obtained SAXS patterns (Fig. 3a) and the Porod exponent (PE) related to the fractal dimension, in both low and high q-range, the Porod regime can be determined (Ruland 2001) following the relation I(Q) ~ q^(−PE)^, where 1 < (PE) < 3 is attributed to mass fractals and 3 < (PE) < 4 to surface fractals. Mass fractals are usually found for interlinked chains (PE = 2) or clustered networks (PE = 3), while surface fractals are attributed to rough surfaces (PE = 3) or smooth surfaces (PE = 4). According to the exponent of the scattering curve in the low q-range the aerogel samples CL-TBAF, CL-EMIm and CL-NMMO were characterized as mass fractals, rather interlinked chains, whereas the high value of the samples CL-CTO distinguished the sample as either a clustered network or a rough surface fractal. Furthermore a PE value towards 3 can be interpreted as related to a 3D-branched structure.

For CL-NMMO and CL-CTO aerogels, a higher value of R_c_, together with the increased skeletal density and crystallinity values, corresponds with the good dimensional and mechanical stability and somewhat lower inner surface areas. Especially CL-CTO aerogels (cellulose II crystallinity 46–50 %) tend towards a more compact, 3D-branched structure as shown by PE, which most likely contributes also to the comparable high specific modulus (*E*_ρ_). In contrast, the ductile CL-EMIm aerogels feature a smaller value of R_c_, low crystallinity (13 %) and a lower PE pointing towards 2-dimensional fractal agglomerates with rougher surfaces.

Taking the measured porosity values (Table 5) into account, the fractions ϕ_1_ and ϕ_2_ = 1 − ϕ_1_ could be calculated for the aerogels which allowed the determination of the inner surface area-to-volume ratio of pores (S/V) by SAXS in addition to the SA_V_ parameter derived from the N_2_ adsorption/desorption experiments (Table 4). The S/V and SAv values show the same trend while they differ by factors of 4–5. The reason for the observed differences is not yet clear, cannot be explained solely by the fact that SAXS includes the surface of closed pores as well, and will be subject of further investigations. The loose packing of the cellulose fibrils in highly amorphous CL-TBAF and CL-EMIm aerogels resulted in increased specific surface areas (SSA) as well as surface area-to-volume ratios (SA_V_). The nanostructured fibril network of the TBAF/DMSO derived sample (R_c_ = 4.0 nm) exhibited the highest SSA of 328 m^2^ g^−1^. Conversely, the tight packing of the ordered structures in CL-CTO aerogels resulted in decreased accessible surface areas. The low SAV of CL-CTO (5.7 m^2^ cm^−3^) is furthermore a consequence of the low cellulose concentration of the respective Ca(SCN)_2_·8H_2_O/LiCl solution (1.5 w%) and can therefore not directly compared with the SAV values of the other samples as pore size and pore walls may change as a function of cellulose concentration using the same solvent.

**Table 4:** Specific surface area (SSA), surface area-to-volume ratio (SA_V_) and C constant obtained by nitrogen sorption experiments at 77 K and inner surface area-to-volume ratio (S/V) from SAXS of aerogels derived from solutions of cotton linters (CL) in TBAF/DMSO (CL-TBAF), [EMIm][OAc]/DMSO (CL-EMIm), NMMO·H_2_O (CL-NMMO) and Ca(SCN)_2_·8H_2_O/LiCl (CL-CTO)

Sample name	CL-TBAF	CL-EMIm	CL-NMMO	CL-CTO
CL (w%)	3.0	3.0	3.0	1.5
SSA (m^2^ g^−1^)	328 ± 1 (10)	246 ± 2 (5)	250 ± 10^a^	190 ± 4 (5)
SA_V_ (m^2^ cm^−3^)	22.5 ± 0.1 (10)	13.7 ± 0.1 (5)	15.7 ± 0.6^a^	5.7 ± 0.1 (5)
C	161 ± 6 (10)	165 ± 22 (5)	126 ± 10 (10)	125 ± 27 (5)
S/V (m^2^ cm^−3^)	112 ± 1	74 ± 3	74 ± 2	21.4 ± 0.6

The respective number of samples is given in brackets

^a^Data obtained from Liebner et al. (2009)

#### Molar mass distribution

**Fig. 4:**
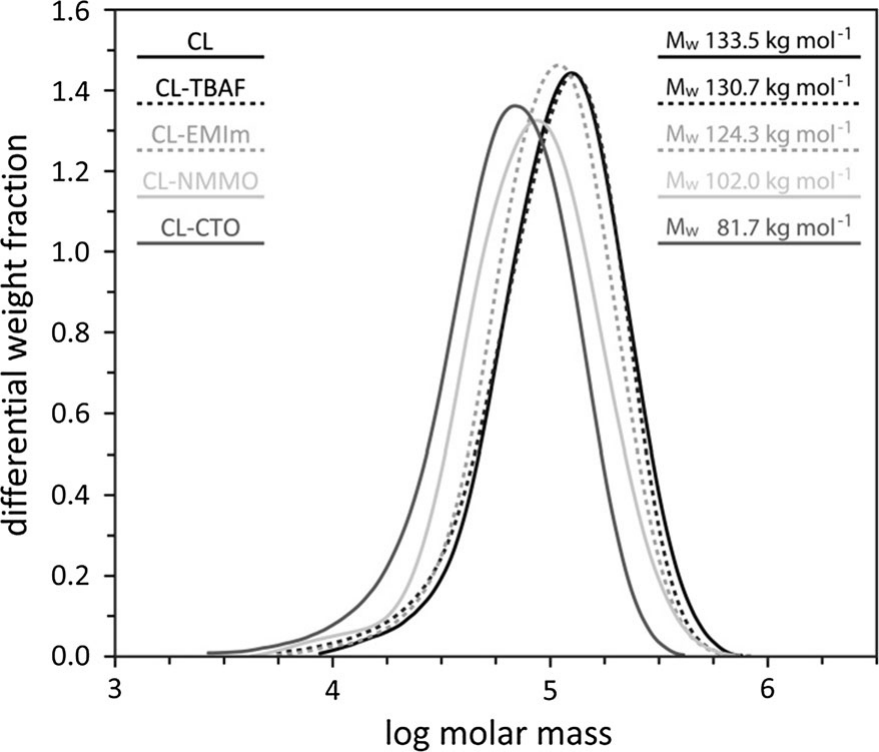
Molecular weight distribution and weight average molecular weight (Mw) of the starting material (*CL* cotton linters) and aerogels derived from solutions of CL in TBAF/DMSO (CL-TBAF), [EMIm][OAc]/DMSO (CL-EMIm), NMMO·H_2_O (CL-NMMO) and Ca(SCN)_2_·8H_2_O/LiCl (CL-CTO)

Cellulose integrity strongly depended on the respective dissolution method (Fig. 4). The weight average molecular weight (M_w_) of CL-TBAF and CL-EMIm aerogels was hardly affected compared to the starting material (cotton linters, CL). Both solvents had been applied at comparatively low temperatures of 60 °C (CL-TBAF) and room temperature (CL-EMIm). In contrast, the M_w_ of CL-NMMO (dissolution at 110 °C) and CL-CTO (140 °C) were 24 and 39 %, respectively, below the M_w_ of CL.

Regarding solutions of CL in NMMO, a previous study showed that the shift of the distribution towards lower molecular weights is connected to the formation of new carbonyl groups, indicating that the main degradation mechanism in stabilized Lyocell dopes is beta-alkoxy elimination (Liebner et al. 2009). Kuga (1980) prepared cellulosic lyogels from 59 w% calcium thiocyanate aqueous solutions (molar ratio H_2_O:Ca^2+^ = 6:1) of Whatman CF-1 cellulose powder (DP 180), dissolving sulfite pulp (DP 770) and cotton linters (DP 1620) and reported a considerable loss in DP (CF-1: 6 %; dissolving pulp: 36 %; CL: 69 %). However, since the elemental composition of the coagulated cellulose samples was in agreement with that of the respective starting material, chemical derivatization of cellulose was ruled out as a possible reason for cellulose degradation, but assigned to the high input of thermal energy during dissolution (Kuga 1980).

### Porosity characteristics

**Fig. 5:**
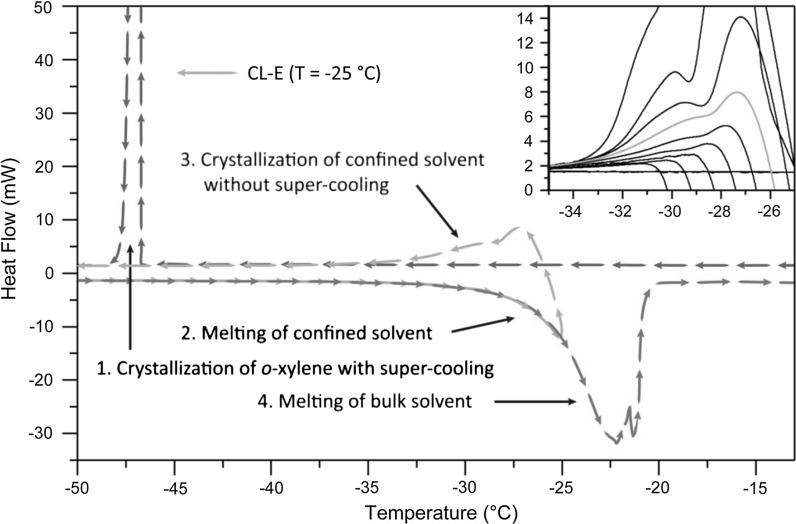
DSC thermogram of a CL-EMIm aerogel soaked in *o*-xylene—the crystallization curve (*3*) acquired after heating from −70 to −25 °C (*2*) was used to calculate the PSD. *Inset* selection of 11 thermograms measured on CL-EMIm aerogels, amplified in the exothermic peak (*3*) associated with the crystallization of the confined solvent

A thermogram obtained by thermoporosimetry for sample CL-EMIm is illustrated in Fig. 5 (and in Fig. S4 co-displayed with a thermogram of neat o-xylene; supplementary information), showing the following characteristic regions and thermal events:

- The exothermic peak observed during the first cooling step (T = −47 °C) results from the crystallization of *o*-xylene accompanied by a super-cooling phenomenon.
- When increasing the temperature from −70 up to −25 °C, an endothermic peak in the higher temperature range indicates melting of the solvent confined in pores below about 10 µm of the aerogel.
- Heating is stopped at −25 °C, a temperature which was chosen to avoid melting of the bulk solvent (Fig. 5, Inset), and the system is slowly cooled down for a second cycle without super-cooling, as the bulk solvent already exists in solid state. The exothermic peak pattern now observed at around T = −29 °C is solely caused by crystallization of the confined solvent. Deconvolution of the peak pattern in this range using a pseudo-Voight function with least-square refinement, and subsequent integration of the resolved peaks can provide detailed information regarding pore size distribution, their modality, and pore geometry.
- Final heating from T = −70 °C up to room temperature causes a broad endothermic peak at approx. 22 °C associated to the melting of the bulk solvent.

**Fig. 6:**
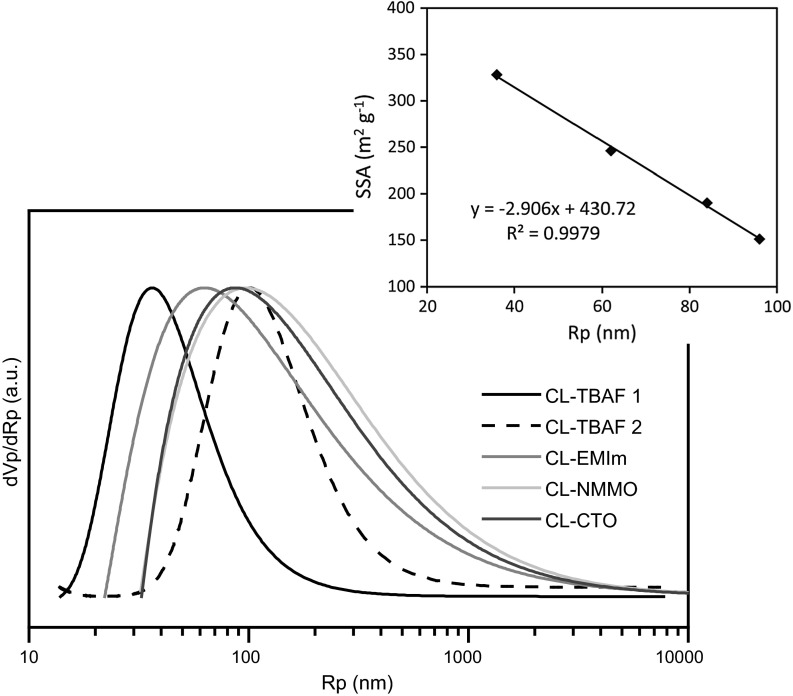
Normalized pore size distribution (PSD) as obtained by TPM analysis. Aerogels have been derived from solutions of 3.0 w% cotton linters in TBAF/DMSO (CL-TBAF), [EMIm][OAc]/DMSO (CL-EMIm), NMMO·H_2_O (CL-NMMO) and 1.5 w% cotton linters in Ca(SCN)_2_·8H_2_O/LiCl (CL-CTO). *Inset* Correlation between specific surface area (SSA) and peak maxima of the PSDs

The PSDs of all aerogels prepared in this study (Fig. 6) were calculated by transforming the respective thermograms, i.e. the crystallization curves acquired after heating from −70 to −25 °C, according to Eq. (9). The margin of error of TPM measurements, arising mainly from the TPM calibration curves, ranges from 2 to 9 % for macropores and is less than 5 % for mesopores (Dessources et al. 2012).

The structure of cellulosic aerogels consists of a cellulose fibril network and an interstitial hierarchical system of interconnected voids which often comprises more than 95 % of the sample volume. Due to the random character of the fibril network, a narrow PSD is quite unlikely, especially at such low aerogel densities. Indeed, in case of the investigated cellulose II aerogels, broad PSDs have been obtained, covering pore sizes from the nano- to the micrometer range. The respective peak position of the pore radius and the curve shape showed a considerable influence of the applied solvent system. Loosely packed, amorphous aerogels were found to tend towards smaller pore sizes, especially the TBAF/DMSO derived aerogel (CL-TBAF), which, in agreement with SEM analysis (Fig. 1), had a comparatively narrow PSD with an upper limit at around 1 µm. CL-TBAF aerogels showed an additional larger pore fraction peaking at 101 nm corresponding to the cracks visible in the SEM micrographs.

Irrespective of the type of solvent used, the specific surface area (SSA) calculated from nitrogen sorption isotherms (Fig. S1–S3, supplementary information) showed a linear negative correlation to the peak pore size of the cellulosic aerogels as determined by TPM (Fig. 6, inset). Considering the strong structural differences between the samples, the low deviation of the values from this trend is remarkable. Provided thorough calibration, this dependency could even be used to estimate the pore size of this particular type of aerogels from SSA, and vice versa.

The overall porosity was around 96 % for aerogels of comparable cellulose content (3 w % CL) and 98 % in case of CL-CTO (1.5 w% CL) (Table 5).

**Table 5:** Average pore size (Ø) according to thermoporosimetry analysis and nitrogen sorption experiments at 77 K, pore volume, and overall porosity as obtained by helium pycnometry for aerogels derived from solutions of cotton linters in TBAF/DMSO (CL-TBAF), [EMIm][OAc]/DMSO (CL-EMIm), NMMO·H_2_O (CL-NMMO) and Ca(SCN)_2_·8H_2_O/LiCl (CL-CTO)

Sample name	CL-TBAF	CL-EMIm	CL-NMMO	CL-CTO
CL (w%)	3.0	3.0	3.0	1.5
Pore size TPM (nm)	36/101	62	96	84
Modal pore size BJH (nm)	34.5	34.4	32.1	2.7
Hydraulic pore size (nm)	72.3	38.4	36.7	23.6
Pore volume (cm^−3^ g^−1^)	5.9	2.4	1.4	0.8
Porosity (%)	95.5 ± 0.1	96.3 ± 0.1	95.9 ± 0.1	98.1 ± 0.1

## Conclusions

The type of solvent system used to obtain solutions of cellulose has been demonstrated to have a strong impact on the nanomorphology of cellulose II aerogels derived thereof. This process encompassed (1) coagulation of the biopolymer from solution state by the cellulose antisolvent ethanol and (2) supercritical carbon dioxide extraction of ethanol from the interconnected pore system. Significant differences on the supramolecular and nanostructural level are caused by particular solvent effects and translate into aerogels of comparable density that—although prepared from the same cellulose source and similar concentrations—can considerably differ in their properties, as for example, their mechanical stability.

Significantly increased cellulose II crystallinity was found in aerogels prepared in solvents that allowed the formation of ordered domains or aggregates already prior cellulose coagulation, such as Ca(SCN)_2_·8H_2_O/LiCl (46–50 %) or NMMO·H_2_O (24–27 %). SAXS analysis furthermore revealed that fibrils of 8–9 nm in diameter are the main particles forming the cellulose networks in coagulated cellulose. The skeletal density of the fibril aggregates increased with cellulose crystallinity, which was associated with the presence of thicker nanofibrils, imparting the respective aerogels with good mechanical properties. According to the fractal dimension of the SAXS patterns, aerogels derived from Ca(SCN)_2_·8H_2_O/LiCl solutions are the only type of cellulose II aerogels in this study which showed surface fractal characteristics indicating 3D-branched structures. This explains the excellent mechanical and dimensional stability, but somewhat lower surface area of these aerogels. All other cellulose solvent systems afforded aerogels of mass fractal characteristics, suggesting a cellulose agglomerate structure consisting of disc-like shaped particles of less entanglement, hence poorer mechanical properties. Interestingly, aerogels derived from cellulose solutions in TBAF/DMSO deviated somewhat from all other aerogels. Regardless of their entirely amorphous cellulose structure, CL-TBAF samples featured the highest rigidity under compressive stress. It is assumed this is due to their uniform morphology, small diameter of fibrils forming the scaffold, and small size of pores hosted by the cellulose network. In accordance, the determination of the accessible surface of open pores by nitrogen sorption (328 m^2^ g^−1^) as well as of the total pore surface area by SAXS (112 m^2^ cm^−3^) revealed a comparatively high internal surface of CL-TBAF aerogels. Samples which had higher crystallinities and larger nanofibril diameters (e.g. CL-CTO) generally tended towards lower surface areas. TPM which was adapted for the use on cellulose-based aerogels in course of this work, revealed broad pore size distributions which exhibited a shift of the position of the main peak (36–96 nm) towards smaller pores with increasing specific surface area of the cellulosic aerogel sample, irrespective of the applied solvent.

The results obtained in this study show:

- Thermoporosimetry is a suitable tool to determine the pore size determination of cellulose based aerogels.
- A clear relationship between solvent system, achieved structure and mechanical properties of the cellulosic aerogels.
- Careful selection of the solvent system allows tailoring cellulose II aerogel properties.

Thus this study supports the further development of cellulosic aerogels for high-performance applications, such as thermal and acoustic insulation, cell scaffolding materials for tissue engineering, or matrix materials for 3D display applications, by choosing a suitable solvent system to achieve the desired nanostructure and material properties.

## Electronic supplementary material

Below is the link to the electronic supplementary material.

Supplementary material 1 (DOCX 153 kb)
