# Supplementary material for: Impact of selected solvent systems on the pore and solid structure of cellulose aerogels
Source: Cellulose (Lond). 2016 Mar 7;23:1949–66. doi: 10.1007/s10570-016-0896-z (PMC4869744; doi:10.1007/s10570-016-0896-z)
Supplement: Supplementary file 1 — Supplementary material 1 (DOCX 153 kb) [file 10570_2016_896_MOESM1_ESM.docx]

# Impact of selected solvent systems on the pore and solid structure of cellulose aerogels

Nicole Pircher^a^, Leticia Carbajal^b,c^, Christian Schimper^a^, Markus Bacher^a^, Harald Rennhofer^d^, Jean-Marie Nedelec^b,c^, Helga C. Lichtenegger^d^, Thomas Rosenau^a^, Falk Liebner^a,*^

^a^ University of Natural Resources and Life Sciences Vienna, Division of Chemistry of Renewables, Konrad-Lorenz-Straße 24, A-3430 Tulln, Austria

^b^ Clermont Université, Ecole Nationale Supérieure de Chimie de Clermont-Ferrand, Institute of Chemistry of Clermont-Ferrand, BP 10448, 63000, Clermont-Ferrand, France

^c^ Centre National de la Recherche Scientifique, UMR 6296, Institute of Chemistry of Clermont-Ferrand, 24 Avenue des Landais, 63171 Aubière, France

*^d^ University of Natural Resources and Life Sciences Vienna, Institute of Physics and Material Sciences, Peter Jordan Straße 82, A-1190 Wien, Austria*

* corresponding author: falk.liebner@boku.ac.at

Supplementary Information

Figure S1: Nitrogen sorption (77K) of aerogels obtained by coagulation of cellulose from different solvent systems

Figure S2: Desorption branch of the nitrogen sorption isotherms (77K) of aerogels obtained by coagulation of cellulose from different solvent systems

Figure S3: Desorption branch of the nitrogen sorption isotherms (77K) of aerogels obtained by coagulation of cellulose from different solvent systems (zoom of the pore size range between 10 and 100 Å)

Figure S4: Thermogram of neat o-xylene (dotted line) in comparison to the thermogram of a CL-EMIm aerogel sample (continuous line).
